# Supplementary figures and images for: Gait outcomes of older adults receiving subacute hospital rehabilitation following orthopaedic trauma: a longitudinal cohort study
Source: BMJ Open. 2017 Jul 20;7(7):e016628. doi: 10.1136/bmjopen-2017-016628 (PMC5541473; doi:10.1136/bmjopen-2017-016628)

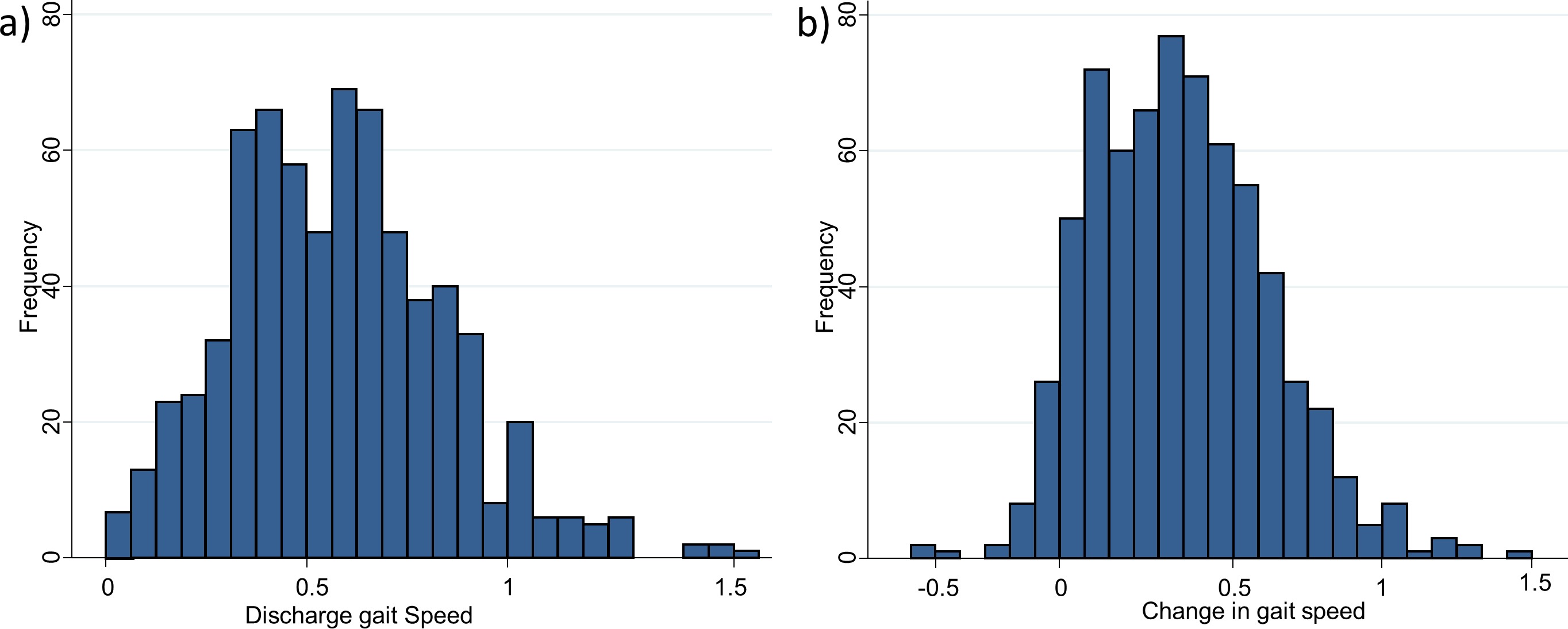

Supplement: Supplementary file 1 [file bmjopen-2017-016628supp001.jpg]
