# Supplementary material for: Gait outcomes of older adults receiving subacute hospital rehabilitation following orthopaedic trauma: a longitudinal cohort study
Source: BMJ Open. 2017 Jul 20;7(7):e016628. doi: 10.1136/bmjopen-2017-016628 (PMC5541473; doi:10.1136/bmjopen-2017-016628)
Supplement: Supplementary file 2 [file bmjopen-2017-016628supp002.pdf]

Supplementary Table S1

Sensitivity analyses coefficients from two generalized linear models examining the association of patient and clinical factors with a) change in gait speed and b) gait speed at discharge

**Model dependent variable**	**Independent variables**	**Coefficient × 10**	**95% Confidence Interval**		***p* value**
Discharge gait speed	Age	-0.01	-0.03	0.00	0.24
	Male gender	0.54	0.14	0.95	< 0.01
	Length of stay	-0.01	-0.06	0.08[a]	0.76
(Wald chi square(12)= 241.58, p < 0.001)	Type of injury				
	Neck of femur	Referent			
	Spinal	0.13	-0.34	0.61	0.58
	Pelvic	0.69	0.12	1.25	< 0.01
	Upper limb	-0.32	-0.91	0.26	0.27
	Multiple	1.08	0.28	1.89	< 0.01
	Femoral	-0.16	-0.83	0.50	0.62
	Lower Limb	0.18	-0.79	1.17	0.71
	Other axial	0.15	-0.73	1.10	0.73
	Gait speed	4.54	3.81	5.27	<0.001
	Use of mobility aid	-0.95	-1.86	-0.04	0.03
Change in gait speed	Age	-0.01	-0.03	0.00	0.14
	Male gender	0.51	-0.00	0.87	< 0.01
	Length of stay	0.04	-0.06	0.08[a]	0.91
(Wald chi square(12)= 344.77, p < 0.001)	Type of injury				
	Neck of femur	Referent			
	Spinal	-0.13	-0.34	0.61	0.58
	Pelvic	0.69	0.12	1.25	< 0.01
	Upper limb	-0.32	-0.91	0.26	0.27
	Multiple	1.08	0.28	1.89	< 0.01
	Femoral	-0.16	-0.83	0.50	0.62
	Lower Limb	0.18	-0.79	1.17	0.71
	Other axial	0.15	-0.73	1.04	0.73
	Gait speed	-5.45	-6.18	-4.72	<0.001
	Use of mobility aid	-0.95	-1.86	0.04	0.03

a= coefficients have been multiplied x 10^2
