# Supplementary material for: Gait outcomes of older adults receiving subacute hospital rehabilitation following orthopaedic trauma: a longitudinal cohort study
Source: BMJ Open. 2017 Jul 20;7(7):e016628. doi: 10.1136/bmjopen-2017-016628 (PMC5541473; doi:10.1136/bmjopen-2017-016628)
Supplement: Author's manuscript [file bmjopen-2017-016628.draft_revisions.pdf]

BMJ Open

# Gait outcomes of older adults receiving subacute hospital rehabilitation following orthopedic trauma

| | |
|---|---|
| Journal: | *BMJ Open* |
| Manuscript ID | bmjopen-2017-016628 |
| Article Type: | Research |
| Date Submitted by the Author: | 02-Mar-2017 |
| Complete List of Authors: | Mathew, Saira; Queensland University of Technology, Institute of Health and Biomedical Innovation<br>Varghese, Paul; Princess Alexandra Hospital, Geriatric Assessment and Rehabilitation Unit; University of Queensland , School of Medicine<br>Kuys, Suzanne; Metro North Hospital and Health Service, Allied Health Research Collaborative; Australian Catholic University, School of Physiotherapy<br>Heesch, Kristiann; Queensland University of Technology, Institute of Health & Biomedical<br>McPhail, Steven; Queensland University of Technology, Institute of Health and Biomedical Innovation; Metro South Hospital and Health Service, Centre for Functioning and Health Research |
| <b>Primary Subject Heading</b>: | Geriatric medicine |
| Secondary Subject Heading: | Public health, Rehabilitation medicine |
| Keywords: | Gait, fracture, geriatric, rehabilitation, velocity |
| | |

SCHOLARONE™
Manuscripts

For peer review only - http://bmjopen.bmj.com/site/about/guidelines.xhtml

# Gait outcomes of older adults receiving subacute hospital rehabilitation following orthopedic trauma

Saira A. Mathew, MHP[1, 2], Paul Varghese, MBBS[3, 4], Suzanne S Kuys, PhD[5], Kristiann C. Heesch, DrPH[1], and Steven M. McPhail, PhD[1, 2*]

[1] School of Public Health & Social Work and Institute of Health and Biomedical Innovation, Queensland University of Technology, Brisbane, Australia, [2] Centre for Functioning and Health Research, Metro South Health, Brisbane, Australia, [3] School of Medicine, The University of Queensland, Australia, [4] Geriatric Assessment and Rehabilitation Unit, Princess Alexandra Hospital, Brisbane, Australia, [5] School of Physiotherapy, Australian Catholic University, Brisbane, Australia

*Corresponding Author:

Attention: Associate Professor Steven McPhail

Email: steven.mcphail@qut.edu.au

Mail: c/o ABIOS / CFAHR, PO Box 6053, Buranda 4102, Queensland, Australia

Telephone: +61 7 3406 2266

Facsimile: +61 7 3406 2267

Alternate Corresponding Author: Saira Mathew

Email: sairaannmathew2@qut.edu.au

Running head: Gait speed after orthopedic trauma

Abstract word count – 300

Main text word count – 2867

For peer review only

## Abstract

**Objectives:** This study aimed to describe gait speed at admission and discharge from inpatient hospital rehabilitation among older adults recovering from orthopedic trauma, and factors associated with gait speed performance and discharge destination.

**Design:** A longitudinal cohort study was conducted**.**

**Setting:** Australian tertiary hospital subacute rehabilitation wards.

**Participants:** Patients aged >=60 years recovering from orthopedic trauma (n=746, 71% female) were eligible for inclusion.

**Interventions:** Usual care (multidisciplinary inpatient hospital rehabilitation).

**Primary and secondary outcome measures:** Gait speed was assessed using the timed 10m walk test. The proportion of patients exceeding a minimum gait speed threshold indicator (a-priori 0.8m/s) of community ambulation ability was calculated. Generalized linear models were used to examine associations between patient and clinical factors with gait speed performance and being discharged to a residential aged care facility.

**Results:** At discharge, 18% of patients (n=135) exceeded the 0.8m/s threshold indicator for community ambulation ability. Factors associated with faster gait speed at discharge included male sex (*B*=0.43, 95% CI -0.01 to 0.87), admitted for pelvic (*B*=0.76, 95% CI 0.14 to 1.37) or multiple fractures (*B*=1.13, 95% CI 0.25 to 2.01) (versus hip fracture), not requiring a mobility aid (*B*=-0.93, 95% CI -1.89 to 0.01), and faster admission gait (*B*=5.77, 95% CI 5.03 to 6.50). Factors associated with being discharged to residential aged care included older age (OR=1.06, 95% CI 1.03 to 1.10), longer length of stay (OR=1.01, 95% CI 1.01 to 1.02), admitted for upper limb fracture (versus hip fracture) (OR=2.81, 95% CI 1.32 to 5.97) and lower Functional Independence Measure cognitive score (OR=0.89, 95% CI 0.86 to 0.92).




For peer review only - http://bmjopen.bmj.com/site/about/guidelines.xhtml

**Conclusions:** Patients with a range of injury types, not only those presenting to hospital with hip fractures, are being discharged with slow gait speeds that are indicative of limited functional mobility and a high risk of further adverse health events.

**Keywords**: Gait, fracture, geriatric, rehabilitation, velocity

For peer review only

**Article summary**

**Strengths and limitations of this study**

- The study examined discharge gait speeds and discharge destinations of older adults admitted for subacute inpatient hospital rehabilitation for a range of orthopedic injury types.
- The inclusion of all admissions meeting pre-specified inclusion criteria reduced the risk of sampling bias.
- This study was conducted at a single subacute hospital rehabilitation unit and findings may not be generalized to dissimilar clinical settings or healthcare systems.

## Introduction

Orthopedic injuries associated with age-related frailty carry a substantial health and quality of life burden for older adults, and consume considerable healthcare resources[1, 2]. Older adults who sustain orthopedic trauma may experience difficulty returning to premorbid functional status, and participating in activities of daily living[3]. The risk of disability, post-surgical complications, and subsequent fractures increases with older age[4]. Older adults who have been hospitalized with a fragility fracture may require additional supports or admission to a residential aged care facility[3].

To reduce the risk of requiring admission to an aged care facility and to optimize functional recovery, many patients receive inpatient hospital rehabilitation[5]. In a hospital rehabilitation setting, mobility is frequently evaluated through performance-based measures such as gait speed assessment[6, 7]. Gait is a key rehabilitation outcome and a determinant of discharge destination. Slow gait speed has been associated with disability risk, hospitalization and premature mortality, and improvements in gait speed have been associated with lower risk of mortality and independence in community ambulation[6-9]. An ability to walk 46 m at a speed between 0.9m/s and 1.2m/s has been reported to be the minimum requirement for older adults to ambulate independently in the community[10]. However, some researchers propose that a gait speed of 0.8m/s is the minimum required to ambulate independently and participate in community activities[10, 11]. Gait speeds of 0.4-0.8m/s have been associated with independently performing activities of daily living, but with limited community ambulation[11, 12]. Individuals with gait speeds <0.4m/s are likely to require assistance with performing some household activities and require full assistance (e.g., wheelchair use) for community activities.

For peer review only

Previous studies have reported estimates of clinically important differences in gait speed during the first year of recovery from a fracture and the characteristics of independent community ambulators[7, 13, 14]. Small, but meaningful improvements in gait speed, 0.10 to 0.17m/s, have been observed during recovery from hip fractures, and substantial improvements, ranging from 0.17 to 0.26m/s, have also been observed[14]. While proficient motor control and sound cognitive function have been associated with independent community ambulation, few studies have reported factors associated with changes in gait speed and discharge destination among older adults with orthopedic trauma related injuries[7, 13, 14].

This study had three objectives to be investigated in a sample of older adults admitted to a subacute hospital rehabilitation unit with orthopedic trauma related injuries. The first was to describe gait performance at admission and discharge from hospital rehabilitation. The second objective was to calculate the proportion of the sample who met a minimum gait speed threshold indicator for community ambulation at discharge and MCID (minimal clinically important difference) for change in gait speed during inpatient hospital rehabilitation. The third objective was to examine which patient and clinical factors were associated with a) change in gait speed, b) gait speed at discharge and c) discharge to a residential care facility (interim care, hostel or residential aged care facilities).

## Methods

### Design

A longitudinal cohort study with two assessment points was conducted.

### Participants and settings

For peer review only

Participants were patients, aged >=60 years, who presented to a tertiary hospital facility with fracture-related injuries that required an inpatient admission. At the participating hospital patients typically receive acute management of their fracture (e.g., reduction and stabilization) and acute inpatient care in a specialized orthopedics unit. Frail older adults who are not able to safely return home following their inpatient admission may receive further subacute inpatient rehabilitation within the hospital. This participating subacute hospital rehabilitation unit was a 76-bed facility providing comprehensive geriatric medicine and multidisciplinary therapy. This included weekday rehabilitative therapies (e.g., physical therapy), nurse supervised mobility (e.g., walking from the bedside to a ward-based dining area for meals for patients with appropriate mobility) and weekly case conferencing within the multidisciplinary team.

Patients were not eligible for inclusion if they were admitted for non-orthopedic traumatic injuries or for injuries that included serious neurological insults (e.g., spinal cord injury), or were transferred to another hospital without completing subacute inpatient hospital rehabilitation at the participating facility. Patients who died during their inpatient stay were also excluded.

**Procedures**

Patients admitted for subacute inpatient hospital rehabilitation at the participating hospital routinely complete assessments within 72 hours of admission and prior to discharge. As part of these two assessments, gait performance was assessed by their treating physiotherapists using the timed 10m walk test[15-17].

*Gait speed assessment*

For peer review only

The timed 10m walk test was used to assess patients' walking ability over 14m, with the middle 10m timed using a stopwatch. In the present study, treating physiotherapists followed standardized testing procedures[15-17], including instructions to walk at a comfortable and safe pace and permitting the use of a mobility aid (e.g., a walking frame). Patients unable to complete the test without physical assistance of another person were considered unable to complete the test.

*Clinical and patient characteristics*

Clinical and patient characteristics collected at admission included age, sex and type of injury(s). At admission and discharge from the subacute inpatient rehabilitation unit, the use of a mobility aid was recorded, and two functional measures were completed: the Functional Independence Measure (FIM motor and FIM cognitive) by an occupational therapist trained in the FIM assessment procedures, and the Modified Elderly Mobility Scale by a physiotherapist[17, 18]. At discharge, the length of stay in the hospital rehabilitation unit was recorded. Three rehabilitation outcomes were of interest for this study; change in gait speed (between the admission and discharge assessment), gait speed at discharge and discharge to a residential aged care facility.

**Statistical Analysis**

For the purpose of describing gait performance, participants were classified into one of four categories: a) unable to complete the timed 10m walk test at admission and discharge; b) unable to complete the timed 10m walk test at admission, but able at discharge c) able to complete the test at admission and discharge, and d) able to complete the test at admission, but unable at discharge. Frequency histograms examined the distribution of discharge gait speed and change in gait speed after excluding patients unable to complete the assessments at

For peer review only

admission and discharge. Frequencies (and proportion) of patients who met the previously reported minimum threshold gait speed of 0.8m/s[16] which is indicative of ability to successfully ambulate in the community, were calculated. Also calculated were the frequencies and proportions of patients who experienced a change in gait speed that exceeded a previously reported estimate of MCID of 0.10m/s [19].

Three generalized linear models were used to examine patient and clinical characteristics associated with a) change in gait speed, b) discharge gait speed and c) whether or not patients were discharged to a residential aged care facility. The Gaussian family and identity link option was used for both gait speed models, while the binomial family and logit link option was used for the model examining discharge to a residential aged care facility. Patient and clinical characteristics included as independent variables in all models were patient age (continuous, in years), sex, length of stay (continuous, in days), type of injury (nine categories), admission gait speed (continuous, in m/s), and use of a mobility aid at admission (no/yes). In the discharge destination model three additional variables were included: admission Modified Elderly Modified Scale (MEMS) score, admission FIM cognitive score and admission FIM motor score.

Fifty-nine patients (7.9%) had missing data on at least one variable, with missing data considered likely to be missing at random. Multiple imputations by chained equations were used to enable inclusion of these patients in the modeling[20]. Multicollinearity was assessed using variance inflation factors and tolerance statistics. All were <5, indicating multicollinearity was not present. Robust standard errors (Huber-White Sandwich Variance Estimator approach) were used to account for potential heteroscedasticity[21, 22].

For peer review only

Sensitivity analyses were conducted for the two generalized linear models that examined patient and clinical characteristics associated with a) change in gait speed, b) discharge gait speed. These sensitivity analyses excluded patients assigned a gait speed of 0m/s for the primary analyses due to being unable to complete the timed 10m walk test at both admission and discharge assessments. This was conducted to examine whether the exclusion of this subset of patients influenced the findings of the aforementioned linear models. All analyses were conducted using Stata 13 (StataCorp LP, College Station, TX, USA) and alpha was set at a 0.05 significance level.

## RESULTS

A total of 774 patients were initially eligible for inclusion (figure 1). Of these, 28 were not included: 22 (2.8%) patients were transferred to another hospital unit prior to assessment and six (0.8%) died. The characteristics of the remaining 746 patients (96.4%) are presented in table 1. Their improvement in gait speed between admission and discharge is displayed in table 2 and supplementary figure S1. In short, most (76.4%) patients who were able to complete the test at both assessments improved their gait speed by more than MCID in gait of 0.10m/s[19, 23], while just 1.3% decreased by a clinically meaningful margin.

For peer review only

**Table 1: Patient and clinical characteristics**

| Characteristics | | |
|---|---|---|
| Age (mean, SD) | 82 | 8 |
| Gender (n, %) | | |
| Male | 211 | 28.3 |
| Female | 535 | 71.7 |
| Type of injury (n, %) | | |
| Neck of femur | 403 | 54.0 |
| Pelvic | 86 | 11.5 |
| Upper limb | 63 | 8.4 |
| Spinal | 56 | 7.5 |
| Femoral (non-hip) | 41 | 5.5 |
| Multiple | 38 | 5.1 |
| Other axial | 31 | 4.2 |
| Lower limb (not femoral or hip) | 28 | 3.8 |
| Mobility aid at admission assessment (n, %) | | |
| Walking frame (four wheeled walker or hopper) | 611 | 82 |
| Stick (single, two single, four-point) | 49 | 6.6 |
| Wheelchair | 39 | 5.2 |
| Nil | 37 | 4.9 |
| Others(crutches) | 10 | 1.3 |
| Discharge destination (n, %) | | |
| Home | 614 | 82.3 |
| Residential aged care facility (high level care | 78 | 10.4 |
| facility) | 32 | 4.3 |
| Hostel (low level care facility) | 22 | 3.0 |
| Interim | | |
| Length of stay in days (median, IQR) | 35 | 23-56 |
| Mobility aid at discharge assessment (n,%) | | |
| Walking frame (four wheeled walker or hopper) | 448 | 60.2 |
| Stick (single, two single, four-point) | 207 | 27.7 |
| Nil | 62 | 8.3 |
| Wheelchair | 19 | 2.5 |
| Others (crutches) | 10 | 1.3 |

**Table 2: Mean gait speed at admission and discharge**

| | n | Mean gait speed at admission (SD) | Mean gait speed at discharge (SD) |
|---|---|---|---|
| Unable at admission & discharge | 61 | 0 (-) | 0 (-) |
| Unable at admission & able at discharge | 303 | 0 (-) | 0.53 (0.22) |
| Able at admission & unable at discharge | 1 | 0.18 (-) | 0 (-) |
| Able at admission & discharge | 381 | 0.37 (0.21) | 0.64 (0.25) |

- indicates SD not applicable as there was no variation in gait speed for that category

For peer review only

**Factors associated with gait speed at discharge from hospital rehabilitation**

Patients with pelvic fractures ($p<0.01$) and multiple fractures ($p<0.01$) had faster gait speed at discharge than patients with neck of femur fractures (table 3). Compared with female patients, male patients were more likely to have faster gait speed at discharge ($p=0.05$). Also, patients who used a mobility aid at admission were more likely to have walked slower at discharge compared with those who did not use a mobility aid ($p=0.05$).

**Factors associated with change in gait speed during inpatient hospital rehabilitation**

Patients with pelvic fractures ($p<0.01$) and multiple fractures ($p<0.01$) demonstrated greater improvement in gait speed than patients with neck of femur fractures (table 3). Male gender ($p=0.05$) was associated with greater improvement in gait speed, while use of a mobility aid at admission was also likely to be associated with lesser improvement in gait speed ($p=0.05$).

For peer review only

**Table 3: Summary of coefficients from two generalized linear models examining the association of patient and clinical factors with a) change in gait speed b) gait speed at discharge**

| Model dependent variable | Independent variables | Coefficient × 10 | 95% Confidence Interval | | p value |
|---|---|---|---|---|---|
| Discharge gait speed | Age | -0.01 | -0.04 | 0.05[a] | 0.13 |
| | Male gender | 0.43 | -0.01[a] | 0.87 | 0.05 |
| | Length of stay | -0.04[a] | -0.01 | 0.03[a] | 0.26 |
| *(Wald chi square(12)= 362.90, p < 0.001)* | Type of injury | | | | |
| | Neck of femur | Referent | | | |
| | Spinal | 0.09 | -0.43 | 0.62 | 0.73 |
| | Pelvic | 0.76 | 0.14 | 1.37 | < 0.01 |
| | Upper limb | -0.57 | -1.18 | 0.02 | 0.06 |
| | Multiple | 1.13 | 0.25 | 2.01 | < 0.01 |
| | Femoral | -0.29 | -1.06 | 0.47 | 0.45 |
| | Lower limb | 0.68 | -0.31 | 1.69 | 0.17 |
| | Other axial | 0.44 | -0.46 | 1.35 | 0.33 |
| | Gait speed[a] | 5.77 | 5.03 | 6.50 | <0.001 |
| | Use of mobility aid[a] | -0.93 | -1.89 | 0.01 | 0.05 |
| Change in gait speed | Age | -0.01 | -0.04 | 0.05[a] | 0.13 |
| | Male gender | 0.43 | -0.01[a] | 0.87 | 0.05 |
| | Length of stay | -0.04[a] | -0.01 | 0.03 | 0.25 |
| *(Wald chi square(12)= 208.81, p < 0.001)* | Type of injury | | | | |
| | Neck of femur | Referent | | | |
| | Spinal | 0.09 | -0.43 | 0.62 | 0.73 |
| | Pelvic | 0.76 | 0.14 | 1.37 | < 0.01 |
| | Upper limb | -0.57 | -1.18 | 0.02 | 0.06 |
| | Multiple | 1.13 | 0.25 | 2.01 | < 0.01 |
| | Femoral | -0.29 | -1.06 | 0.47 | 0.45 |
| | Lower limb | 0.68 | -0.31 | 1.69 | 0.17 |
| | Other axial | 0.44 | -0.46 | 1.35 | 0.33 |
| | Gait speed[a] | -4.22 | -4.96 | -3.49 | <0.001 |
| | Use of mobility aid[a] | -0.93 | -1.89 | 0.01 | 0.05 |

a= coefficients have been multiplied by100

For peer review only

**Factors associated with discharge to a residential aged care facility**

Older age (p<0.001) and prolonged length of stay (p<0.001) were associated with a lower likelihood of being discharged home (table 4). Participants with upper limb fractures were less likely to be discharged home (p<0.01) than those with neck of femur fractures. A lower

FIM cognitive score was associated with a greater likelihood of being discharged to a residential aged care facility ($p<0.001$).

**Table 4: Summary of odds ratios from a generalized linear model examining the association of patient and clinical factors with discharge to a residential aged care facility**

| Model dependent variable | Independent variables | Odds Ratio | 95% Confidence Interval | | *p* value |
|---|---|---|---|---|---|
| Discharge to a residential aged care facility | Age | 1.06 | 1.03 | 1.10 | < 0.001 |
| | Male gender | 0.98 | 0.58 | 1.67 | 0.95 |
| | Length of stay | 1.01 | 1.01 | 1.02 | < 0.001 |
| | Type of injury | | | | |
| | Neck of femur | Referent | | | |
| *(Wald chi square(15)= 7.84, p < 0.001)* | Spinal | 1.28 | 0.49 | 3.34 | 0.60 |
| | Pelvic | 0.80 | 0.36 | 1.76 | 0.59 |
| | Upper limb | 2.81 | 1.32 | 5.97 | < 0.01 |
| | Multiple | 0.20 | 0.02 | 1.55 | 0.12 |
| | Femoral | 2.02 | 0.85 | 4.82 | 0.11 |
| | Lower limb | 0.83 | 0.22 | 3.10 | 0.79 |
| | Other axial | 0.25 | 0.03 | 2.12 | 0.20 |
| | MEMs score[a] | 0.97 | 0.89 | 1.05 | 0.46 |
| | FIM cognitive score[a] | 0.89 | 0.86 | 0.92 | < 0.001 |
| | FIM motor score[a] | 0.98 | 0.96 | 1.00 | 0.15 |
| | Gait speed[a] | 0.50 | 0.09 | 2.60 | 0.41 |
| | Use of mobility aid[a] | 0.85 | 0.31 | 2.31 | 0.75 |

a=Functional measures assessed at admission

**Sensitivity analyses**

The sensivity analysis findings were consistent with those of the primary analyses of factors associated with discharge gait speed and change in gait speed (supplementary table S1). The coefficient point estimates were similar to the primary analyses, and disrepancies between the primary and sensitivity analysis confidence intervals were of a small magnitude.

For peer review only

## Discussion

This study is the first to examine gait speeds at discharge from subacute hospital rehabilitation in this important clinical group as well as to examine associations between patient and clinical characteristics with gait outcomes and discharge to residential aged care. The gait outcomes ought to be interpreted in the context of the high level of frailty present in the patient sample. The findings could be interpreted as positive with the majority (91.7%) of patients able to walk at least a small distance without assistance of another person at discharge, a commendable achievement given approximately half of the participants were unable to walk without assistance of another person at admission to rehabilitation. Furthermore, a (relatively) low proportion (10.4%) of participants was discharged to a residential aged care facility for ongoing high level nursing care. However, this study also highlighted that only 18% of participants achieved the previously reported 0.8m/s threshold indicator for ability to ambulate in community environments[16].

Previous reports have proposed that gait speeds above 1.0m/s are desirable and those lower than 0.5m/s are associated with poor function and health[24-26]. The slow gait speed observed at completion of inpatient rehabilitation in the present study is not an indictment of the inpatient model of care at the participating facility. It was encouraging that over three quarters of participants could be considered to have improved their gait speed by a clinically meaningful margin during their stay. The mean gait speed at discharge was consistent with a systematic review of prior reports from similar subacute hospital settings that included non-orthopedic patients[27]. However, the findings highlight the importance of planning for an integrated transition from hospital to community-based services in the initial post-hospitalization period for older people recovering from orthopedic trauma. This may include engagement with community-based therapies for patients who have potential to extend gains attained during inpatient rehabilitation, as well as supportive care interventions that seek to

For peer review only - http://bmjopen.bmj.com/site/about/guidelines.xhtml

maximize patients' quality of life and mitigate risk of adverse events. Prior research has highlighted the potential effectiveness (and cost-effectiveness) of community-based interventions for frail older adults recovering from injury and illness[28, 29].

Prior studies in the field have typically focused on patients recovering from hip fractures and have often emphasized risk of undesirable outcomes following hip fractures[30-32]. The inclusion of patients recovering from fractures affecting a range of body regions in the present study permitted novel contributions, particularly in the analytical models that examined factors associated with outcomes at discharge from inpatient rehabilitation. For example, patients recovering from upper limb injuries were more likely to be discharged to residential aged care facilities than patients recovering from hip fractures. At first, this finding may seem paradoxical given the extent of prior reporting of challenges and functional deficits faced by people recovering from hip fractures [33, 34], but it may be explained by the likely high level of frailty in patients requiring inpatient hospital rehabilitation following an upper-limb fracture. It is likely that most patients who present to hospital with an upper-limb fracture will be discharged soon after their fracture has been stabilized, and relatively few will require subacute inpatient rehabilitation. Of all older adults who sustain an upper limb injury, it is likely to be the most frail, complex or socially vulnerable who will require subacute inpatient hospital rehabilitation[35]. In comparison, a high proportion of patients who sustain a hip fracture may require subacute hospital rehabilitation regardless of their social circumstances or premorbid functioning to continue rehabilitative therapies in an effort to attain independence with mobility tasks prior to discharge from hospital.

Understanding factors associated with patients experiencing the greatest (or least) improvement in gait speed between admission and discharge offers insights useful for clinical

For peer review only

teams, health service policy related to inpatient rehabilitation and future research in the field. In the present study the greatest improvements in gait speed were observed among patients with pelvic fractures or with multiple traumatic injuries, which was an encouraging finding as patients from these two diagnostic groups have received limited attention in prior research[27, 36]. It was also encouraging that patients with a slow baseline gait speed were among those to demonstrate the greatest improvement in gait speed during their inpatient rehabilitation, perhaps indicative of the fulfilment of (at least theoretical) potential for improvement that accompanies starting from a lower base than others in the cohort. However, not requiring the use of a mobility aid at admission was associated with greater improvement in gait speed. It is plausible that patients not requiring a mobility aid at admission to the rehabilitation unit had lower levels of pre-morbid physical frailty and were more readily able to return to higher gait speeds by discharge than peers in their cohort.

For peer review only

**Strengths and limitations**

A strength of this study was the inclusion of a range of orthopedic injury types present among older adults who were receiving hospital rehabilitation. Second, the inclusion of consecutive admissions meeting the inclusion criteria, with a relatively low rate of missing assessments reduced the risk of sampling bias. One limitation of this approach was that clinical variables were limited to those recorded for all participants during their routine clinical assessment, this meant that height was not available as a potential covariate for adjustment in the generalized linear models. Another important limitation of the study was that it was conducted at a single geographical location in an industrialized nation in a subacute hospital rehabilitation setting. It is possible that older adults recovering from orthopedic injuries in dissimilar societies or healthcare systems may not have had comparable gait performances as patients in the present sample. The study design was appropriate for addressing the intended aims, but it did not


For peer review only - http://bmjopen.bmj.com/site/about/guidelines.xhtml

allow any conclusions to be drawn regarding the effectiveness (or otherwise) of the model of care received by patients in improving gait speed in comparison to other models of care. This topic was considered beyond the scope of the present study, but future investigation of the optimal type and intensity of rehabilitation interventions for improving gait among older adults recovering orthopedic injuries remains a priority for research.

## Conclusion

Most patients were able to complete the 10m walk test without assistance of another person at discharge, but few were likely to be able to ambulate in community environments. The greatest improvements in gait speed were observed among patients with pelvic fractures and those with multiple traumatic injuries. Also noteworthy was that patients receiving hospital-based rehabilitation following injuries affecting upper limbs were more likely to be discharged to residential aged care facilities than patients recovering from hip fractures. Overall, the findings highlight the importance of planning for an integrated transition from hospital to community-based services in the initial post-hospital rehabilitation period. This may include engagement with community-based rehabilitative therapies or supportive care interventions to improve quality of life and mitigate risk of further adverse health events.

For peer review only

## Acknowledgments

*Funding Source*: The study was supported in part by the Health Practitioner Research Support Scheme (Queensland Department of Health). SMM is supported by a National Health and Medical Research Council (of Australia) fellowship

*Author Contributions:* SSK, PV, SMM: obtaining funding and acquisition of data. SAM, SMM, KCH: study design, analysis and interpretation of data. SAM, SMM: drafting of the manuscript. All authors: interpretation of findings, revision of manuscript, approval of final version

*Ethical approval*: The study was approved by the Metro South Human Research Ethics Committee as a low risk investigator initiated study. A waiver of individual consent was granted from the approving ethics committee as assessments were completed as part of routine care, patients did not experience any deviation from their usual clinical care, and the conduct of the study (including data management procedures) preserved patients' rights and welfare.

*No competing interests*: Authors have read and understood BMJ policy on declaration of interests and declare that we have no competing interests.

*Study funding:* The Department of Health Queensland financially supported the study but had no role in the research design, reporting or publishing decision

*Data sharing:* No additional data are available, though details on statistical analysis are available from the corresponding author on request

For peer review only

## References

1. Wiktorowicz M, Goeree R, Papaioannou A, et al. Economic implications of hip fracture: health service use, institutional care and cost in Canada. *Osteoporos Int*2001;**12**(4):271-78

2. Kawryshanker S, Raymond W, Ingram K, et al. Effect of frailty on functional gain, resource utilisation, and discharge destination: an observational prospective study in a GEM Ward. *Curr Gerontol Geriatr Res* 2014;**2014**

3. Pretto M, Spirig R, Kaelin R, et al. Outcomes of elderly hip fracture patients in the Swiss healthcare system. *Swiss Med Wkly* 2010;**140**:w13086

4. Levers MJ, Estabrooks CA, Ross Kerr JC. Factors contributing to frailty: literature review. *J Adv Nurs* 2006;**56**(3):282-91

5. Röder F, Schwab M, Aleker T, et al. Proximal femur fracture in older patients– rehabilitation and clinical outcome. *Age Ageing* 2003;**32**(1):74-80

6. Ostir GV, Berges I, Kuo Y-F, et al. Assessing gait speed in acutely ill older patients admitted to an acute care for elders hospital unit. Arch Intern Med 2012;**172**(4):353-58

7. Hardy SE, Perera S, Roumani YF, et al. Improvement in usual gait speed predicts better survival in older adults. *J Am Geriatr Soc* 2007;**55**(11):1727-34

8. Potter JM, Evans AL, Duncan G. Gait speed and activities of daily living function in geriatric patients. *Arch Phys Med Rehabil* 1995;**76**(11):997-99

9. Purser JL, Weinberger M, Cohen HJ, et al. Walking speed predicts health status and hospital costs for frail elderly male veterans. *J Rehabil Res Develop* 2005;**42**(4):535

10. Andrews AW, Chinworth SA, Bourassa M, et al. Update on distance and velocity requirements for community ambulation. *J Geriatr Phys Ther* 2010;**33**(3):128-34

11. Fritz S, Lusardi M. White paper:"walking speed: the sixth vital sign". *J Geriatr Phys Ther* 2009;**32**(2):2-5

For peer review only

12. Lord SE, McPherson K, McNaughton HK, et al. Community ambulation after stroke: how important and obtainable is it and what measures appear predictive? *Arch Phys Med Rehabil* 2004;**85**(2):234-39

13. Lord SE, Weatherall M, Rochester L. Community ambulation in older adults: which internal characteristics are important? *Arch Phys Med Rehabil* 2010;**91**(3):378-83

14. Alley DE, Hicks GE, Shardell M, et al. Meaningful improvement in gait speed in hip fracture recovery. *J Am Geriatr Soc* 2011;**59**(9):1650-57

15. Stineman MG, Shea JA, Jette A, et al. The Functional Independence Measure: tests of scaling assumptions, structure, and reliability across 20 diverse impairment categories. *Arch Phys Med Rehabil* 1996;**77**(11):1101-08

16. Van Kan GA, Rolland Y, Andrieu S, et al. Gait speed at usual pace as a predictor of adverse outcomes in community-dwelling older people an International Academy on Nutrition and Aging (IANA) Task Force. *J Nutr Health Aging* 2009;**13**(10):881-89

17. Kuys SS, Brauer SG. Validation and reliability of the modified elderly mobility scale. *Australas J Ageing* 2006;**25**(3):140-44

18. Kidd D, Stewart G, Baldry J, et al. The Functional Independence Measure: a comparative validity and reliability study. *Disabil Rehabil* 2009

19. Bohannon RW, Glenney SS. Minimal clinically important difference for change in comfortable gait speed of adults with pathology: a systematic review. J Eval Clini Prac 2014;**20**(4):295-300

20. Royston P, White IR. Multiple imputation by chained equations (MICE): implementation in Stata. *J Stat Softw* 2011;**45**(4):1-20

21. The behavior of maximum likelihood estimates under nonstandard conditions. Proceedings of the fifth Berkeley symposium on mathematical statistics and probability. *Am J Hum Genet* ; 1967.

For peer review only

22. White H. A heteroskedasticity-consistent covariance matrix estimator and a direct test for heteroskedasticity. *Econometrica: J Econometric Soc*1980:817-38

23. Palombaro KM, Craik RL, Mangione KK, et al. Determining meaningful changes in gait speed after hip fracture. *Phys Ther* 2006;**86**(6):809-16

24. Gard SA. Use of quantitative gait analysis for the evaluation of prosthetic walking performance. JPO: *J Prosthet Orthot* 2006;**18**(6):P93-P104

25. Mendelsohn ME, Leidl DS, Overend TJ, et al. Specificity of functional mobility measures in older adults after hip fracture: a pilot study. *Am J Phys Med Rehabil* 2003;**82**(10):766-74

26. Robinett CS, Vondran MA. Functional Ambulation Velocity and Distance Requirements in Rural and Urban Communities A Clinical Report. *Phys Ther* 1988;**68**(9):1371-73

27. Peel NM, Kuys SS, Klein K. Gait speed as a measure in geriatric assessment in clinical settings: a systematic review. *J Gerontol A Bio Sci Med Sci*2013;**68**(1):39-46

28. Leveille SG, Wagner EH, Davis C, et al. Preventing disability and managing chronic illness in frail older adults: A randomized trial of a community-based partnership with primary care. *J Am Geriatr Soc* 1998;**46**(10):1191-98

29. Hausdorff JM, Rios DA, Edelberg HK. Gait variability and fall risk in community-living older adults: a 1-year prospective study. *Arch Phys Med Rehabil* 2001;**82**(8):1050-56

30. Cornwall R, Gilbert MS, Koval KJ, et al. Functional outcomes and mortality vary among different types of hip fractures: a function of patient characteristics. *Clin Orthop Relat Res* 2004(425):64

31. Afilalo J, Eisenberg MJ, Morin J-F, et al. Gait speed as an incremental predictor of mortality and major morbidity in elderly patients undergoing cardiac surgery. *J Am Coll Cardiol* 2010;**56**(20):1668-76

For peer review only

32. Atkinson HH, Rosano C, Simonsick EM, et al. Cognitive function, gait speed decline, and comorbidities: the health, aging and body composition study. *J Gerontol A Bio Sci Med Sci* 2007;**62**(8):844-50

33. Koval KJ, Skovron ML, Aharonoff GB, et al. Ambulatory ability after hip fracture: a prospective study in geriatric patients. *Clin Orthop Relat Res*1995(310):150-59

34. Dennett AM, Taylor NF, Mulrain K. Community ambulation after hip fracture: completing tasks to enable access to common community venues. *Disabil Rehabil* 2012;**34**(9):707-14

35. Lübbeke A, Stern R, Grab B, et al. Upper extremity fractures in the elderly: consequences on utilization of rehabilitation care. *Aging Clin Exp Res*2005;**17**(4):276-80

36. Kwakkel G, Wagenaar RC. Effect of duration of upper-and lower-extremity rehabilitation sessions and walking speed on recovery of interlimb coordination in hemiplegic gait. *Phys Ther* 2002;**82**(5):432-48

For peer review only

## Legends

**Figure 1** Participant flow diagram

**Supplementary figure S1** Frequency of discharge gait speed and change of gait speed

**Supplementary material S1-** Sensitivity analyses coefficients from two generalized linear models examining the association of patient and clinical factors with a) change in gait speed and b) gait speed at discharge

For peer review only

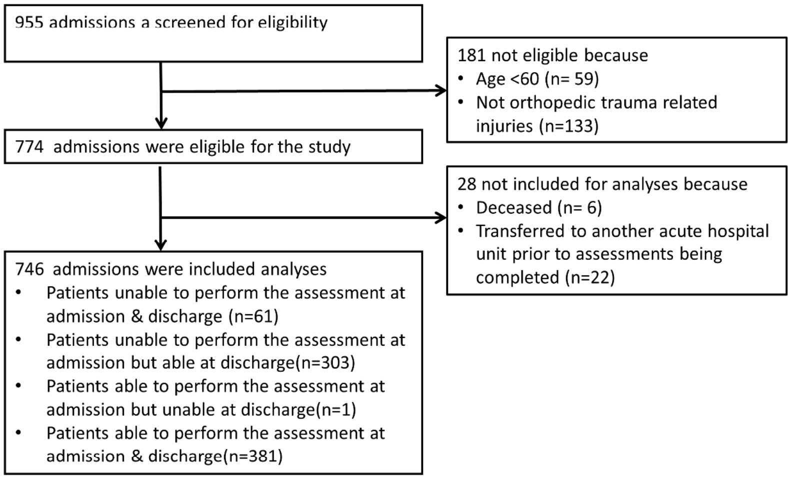


Participant flow diagram

254x190mm (300 x 300 DPI)

For peer review only - http://bmjopen.bmj.com/site/about/guidelines.xhtml

Supplementary Table S1

**Sensitivity analyses coefficients from two generalized linear models examining the association of patient and clinical factors with a) change in gait speed and b) gait speed at discharge**

| Model dependent variable | Independent variables | Coefficient × 10 | 95% Confidence Interval | | *p* value |
|---|---|---|---|---|---|
| Discharge gait speed | Age | -0.01 | -0.03 | 0.00 | 0.24 |
| | Male gender | 0.54 | 0.14 | 0.95 | < 0.01 |
| | Length of stay | -0.01 | -0.06 | 0.08[a] | 0.76 |
| | Type of injury | | | | |
| *(Wald chi square(12)= 241.58, p < 0.001)* | Neck of femur | Referent | | | |
| | Spinal | 0.13 | -0.34 | 0.61 | 0.58 |
| | Pelvic | 0.69 | 0.12 | 1.25 | < 0.01 |
| | Upper limb | -0.32 | -0.91 | 0.26 | 0.27 |
| | Multiple | 1.08 | 0.28 | 1.89 | < 0.01 |
| | Femoral | -0.16 | -0.83 | 0.50 | 0.62 |
| | Lower Limb | 0.18 | -0.79 | 1.17 | 0.71 |
| | Other axial | 0.15 | -0.73 | 1.10 | 0.73 |
| | Gait speed | 4.54 | 3.81 | 5.27 | <0.001 |
| | Use of mobility aid | -0.95 | -1.86 | -0.04 | 0.03 |
| Change in gait speed | Age | -0.01 | -0.03 | 0.00 | 0.14 |
| | Male gender | 0.51 | -0.00 | 0.87 | < 0.01 |
| | Length of stay | 0.04 | -0.06 | 0.08[a] | 0.91 |
| *(Wald chi square(12)= 344.77, p < 0.001)* | Type of injury | | | | |
| | Neck of femur | Referent | | | |
| | Spinal | -0.13 | -0.34 | 0.61 | 0.58 |
| | Pelvic | 0.69 | 0.12 | 1.25 | < 0.01 |
| | Upper limb | -0.32 | -0.91 | 0.26 | 0.27 |
| | Multiple | 1.08 | 0.28 | 1.89 | < 0.01 |
| | Femoral | -0.16 | -0.83 | 0.50 | 0.62 |
| | Lower Limb | 0.18 | -0.79 | 1.17 | 0.71 |
| | Other axial | 0.15 | -0.73 | 1.04 | 0.73 |
| | Gait speed | -5.45 | -6.18 | -4.72 | <0.001 |
| | Use of mobility aid | -0.95 | -1.86 | 0.04 | 0.03 |

a= coefficients have been multiplied x $10^2$

For peer review only

For peer review only

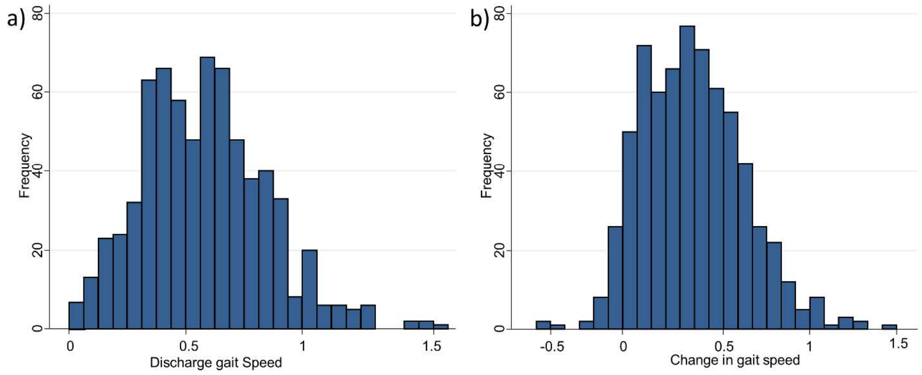


254x190mm (300 x 300 DPI)

**STROBE 2007 (v4) Statement—Checklist of items that should be included in reports of *cohort studies***

| Section/Topic | Item # | Recommendation | Reported on page # |
|---|---|---|---|
| **Title and abstract** | 1 | (*a*) Indicate the study's design with a commonly used term in the title or the abstract | 1-2 |
| | | (*b*) Provide in the abstract an informative and balanced summary of what was done and what was found | 2-3 |
| **Introduction** | | | |
| Background/rationale | 2 | Explain the scientific background and rationale for the investigation being reported | 4-5 |
| Objectives | 3 | State specific objectives, including any prespecified hypotheses | 5 |
| **Methods** | | | |
| Study design | 4 | Present key elements of study design early in the paper | 5 |
| Setting | 5 | Describe the setting, locations, and relevant dates, including periods of recruitment, exposure, follow-up, and data collection | 6 |
| Participants | 6 | (*a*) Give the eligibility criteria, and the sources and methods of selection of participants. Describe methods of follow-up | 6-7 |
| | | (*b*) For matched studies, give matching criteria and number of exposed and unexposed | |
| Variables | 7 | Clearly define all outcomes, exposures, predictors, potential confounders, and effect modifiers. Give diagnostic criteria, if applicable | 7 |
| Data sources/ measurement | 8* | For each variable of interest, give sources of data and details of methods of assessment (measurement). Describe comparability of assessment methods if there is more than one group | 7 |
| Bias | 9 | Describe any efforts to address potential sources of bias | 6 |
| Study size | 10 | Explain how the study size was arrived at | 6 |
| Quantitative variables | 11 | Explain how quantitative variables were handled in the analyses. If applicable, describe which groupings were chosen and why | 7 |
| Statistical methods | 12 | (*a*) Describe all statistical methods, including those used to control for confounding | 7-8 |
| | | (*b*) Describe any methods used to examine subgroups and interactions | 7 |
| | | (*c*) Explain how missing data were addressed | 8 |
| | | (*d*) If applicable, explain how loss to follow-up was addressed | 8 |
| | | (*e*) Describe any sensitivity analyses | 9 |
| **Results** | | | |

For peer review only

| | | | |
|---|---|---|---|
| Participants | 13* | (a) Report numbers of individuals at each stage of study—eg numbers potentially eligible, examined for eligibility, confirmed eligible, included in the study, completing follow-up, and analysed | Figure 1<br>Page 9 |
| | | (b) Give reasons for non-participation at each stage | Figure1 |
| | | (c) Consider use of a flow diagram | Figure 1 |
| Descriptive data | 14* | (a) Give characteristics of study participants (eg demographic, clinical, social) and information on exposures and potential confounders | Table 1<br>Page 9 |
| | | (b) Indicate number of participants with missing data for each variable of interest | Table 1 |
| | | (c) Summarise follow-up time (eg, average and total amount) | Table 1, Page 9 |
| Outcome data | 15* | Report numbers of outcome events or summary measures over time | Table 2. Supplementary figure S1, Page9 |
| Main results | 16 | (*a*) Give unadjusted estimates and, if applicable, confounder-adjusted estimates and their precision (eg, 95% confidence interval). Make clear which confounders were adjusted for and why they were included | Table 3 & Table 4<br>Page 11-13 |
| | | (*b*) Report category boundaries when continuous variables were categorized | |
| | | (*c*) If relevant, consider translating estimates of relative risk into absolute risk for a meaningful time period | |
| Other analyses | 17 | Report other analyses done—eg analyses of subgroups and interactions, and sensitivity analyses | Supplementary table S1, Page 13 |
| **Discussion** | | | |
| Key results | 18 | Summarise key results with reference to study objectives | 14-17 |
| **Limitations** | | | |
| Interpretation | 20 | Give a cautious overall interpretation of results considering objectives, limitations, multiplicity of analyses, results from similar studies, and other relevant evidence | 16 |
| Generalisability | 21 | Discuss the generalisability (external validity) of the study results | 16 |
| **Other information** | | | |
| Funding | 22 | Give the source of funding and the role of the funders for the present study and, if applicable, for the original study on which the present article is based | 18 |

*Give information separately for cases and controls in case-control studies and, if applicable, for exposed and unexposed groups in cohort and cross-sectional studies.

For peer review only

**Note:** An Explanation and Elaboration article discusses each checklist item and gives methodological background and published examples of transparent reporting. The STROBE checklist is best used in conjunction with this article (freely available on the Web sites of PLoS Medicine at http://www.plosmedicine.org/, Annals of Internal Medicine at http://www.annals.org/, and Epidemiology at http://www.epidem.com/). Information on the STROBE Initiative is available at www.strobe-statement.org.

For peer review only

BMJ Open

# Gait outcomes of older adults receiving subacute hospital rehabilitation following orthopedic trauma: a longitudinal cohort study

| | |
|---|---|
| Journal: | *BMJ Open* |
| Manuscript ID | bmjopen-2017-016628.R1 |
| Article Type: | Research |
| Date Submitted by the Author: | 23-May-2017 |
| Complete List of Authors: | Mathew, Saira; Queensland University of Technology, Institute of Health and Biomedical Innovation<br>Varghese, Paul; Princess Alexandra Hospital, Geriatric Assessment and Rehabilitation Unit; University of Queensland , School of Medicine<br>Kuys, Suzanne; Metro North Hospital and Health Service, Allied Health Research Collaborative; Australian Catholic University, School of Physiotherapy<br>Heesch, Kristiann; Queensland University of Technology, Institute of Health & Biomedical<br>McPhail, Steven; Queensland University of Technology, Institute of Health and Biomedical Innovation; Metro South Hospital and Health Service, Centre for Functioning and Health Research |
| <b>Primary Subject Heading</b>: | Geriatric medicine |
| Secondary Subject Heading: | Public health, Rehabilitation medicine |
| Keywords: | Gait, fracture, geriatric, rehabilitation, velocity |
| | |

SCHOLARONE™ Manuscripts

For peer review only

# Gait outcomes of older adults receiving subacute hospital rehabilitation following orthopedic trauma: a longitudinal cohort study

Saira A. Mathew, MHP[1, 2], Paul Varghese, MBBS[3, 4], Suzanne S Kuys, PhD[5], Kristiann C. Heesch, DrPH[1], and Steven M. McPhail, PhD[1, 2*]

[1] School of Public Health & Social Work and Institute of Health and Biomedical Innovation, Queensland University of Technology, Brisbane, Australia, [2] Centre for Functioning and Health Research, Metro South Health, Brisbane, Australia, [3] School of Medicine, The University of Queensland, Australia, [4] Geriatric Assessment and Rehabilitation Unit, Princess Alexandra Hospital, Brisbane, Australia, [5] School of Physiotherapy, Australian Catholic University, Brisbane, Australia

*Corresponding Author:

Attention: Associate Professor Steven McPhail

Email: steven.mcphail@qut.edu.au

Mail: c/o ABIOS / CFAHR, PO Box 6053, Buranda 4102, Queensland, Australia

Telephone: +61 7 3406 2266

Facsimile: +61 7 3406 2267

Alternate Corresponding Author: Saira Mathew

Email: sairaannmathew2@qut.edu.au

Running head: Gait speed after orthopedic trauma

Abstract word count – 300

Main text word count – 2954

For peer review only

**Abstract**

**Objectives:** This study aimed to describe gait speed at admission and discharge from inpatient hospital rehabilitation among older adults recovering from orthopedic trauma, and factors associated with gait speed performance and discharge destination.

**Design:** A longitudinal cohort study was conducted**.**

**Setting:** Australian tertiary hospital subacute rehabilitation wards.

**Participants:** Patients aged >=60 years recovering from orthopedic trauma (n=746, 71% female) were eligible for inclusion.

**Interventions:** Usual care (multidisciplinary inpatient hospital rehabilitation).

**Primary and secondary outcome measures:** Gait speed was assessed using the timed 10m walk test. The proportion of patients exceeding a minimum gait speed threshold indicator (a-priori 0.8m/s) of community ambulation ability was calculated. Generalized linear models were used to examine associations between patient and clinical factors with gait speed performance and being discharged to a residential aged care facility.

**Results:** At discharge, 18% of patients (n=135) exceeded the 0.8m/s threshold indicator for community ambulation ability. Faster gait speed at discharge were found to be associated with being male (*B*=0.43, 95%CI -0.01 to 0.87), admitted with pelvic (*B*=0.76, 95%CI 0.14 to 1.37) or multiple fractures (*B*=1.13, 95%CI 0.25 to 2.01) (versus hip fracture), using no mobility aids (*B*=-0.93, 95%CI -1.89 to 0.01), and walking at a faster gait speed at admission (*B*=5.77, 95%CI 5.03 to 6.50). Factors associated with being discharged to residential aged care included older age (OR=1.06, 95%CI 1.03 to 1.10), longer length of stay (OR=1.01, 95%CI 1.01 to 1.02), having an upper limb fracture (versus hip fracture) (OR=2.81, 95%CI 1.32 to 5.97) and lower Functional Independence Measure cognitive score (OR=0.89, 95%CI 0.86 to 0.92).



For peer review only - http://bmjopen.bmj.com/site/about/guidelines.xhtml

**Conclusions:** Patients with a range of injury types, not only those presenting to hospital with hip fractures, are being discharged with slow gait speeds that are indicative of limited functional mobility and a high risk of further adverse health events.

**Keywords**: Gait, fracture, geriatric, rehabilitation, velocity

**Article summary**

**Strengths and limitations of this study**

- The study examined discharge gait speeds and discharge destinations of older adults admitted for subacute inpatient hospital rehabilitation for a range of orthopedic injury types.
- The inclusion of all admissions meeting pre-specified inclusion criteria reduced the risk of sampling bias.
- This study was conducted at a single subacute hospital rehabilitation unit and findings may not be generalized to dissimilar clinical settings or healthcare systems.

For peer review only

## Introduction

Orthopedic injuries associated with age-related frailty carry a substantial health and quality of life burden for older adults, and consume considerable healthcare resources[1, 2]. Older adults who sustain orthopedic trauma may experience difficulty returning to premorbid functional status, and participating in activities of daily living[3]. The risk of disability, post-surgical complications, and subsequent fractures increases with older age[4]. Older adults who have been hospitalized with a fragility fracture may require additional supports or admission to a residential aged care facility[3].

To reduce the risk of requiring admission to an aged care facility and to optimize functional recovery, many patients receive inpatient hospital rehabilitation[5]. In a hospital rehabilitation setting, mobility is frequently evaluated through performance-based measures such as gait speed assessment[6, 7]. Gait ability is a key rehabilitation outcome, determinant of discharge destination, and limitations in gait may persist at discharge from subacute rehabilitation [8, 9]. Slow gait speed has been associated with disability risk, hospitalization and premature mortality, and improvements in gait speed have been associated with lower risk of mortality and independence in community ambulation [6, 7, 10, 11]. An ability to walk 46 m at a speed between 0.9m/s and 1.2m/s has been reported to be the minimum requirement for older adults to ambulate independently in the community[12]. However, some researchers propose that a gait speed of 0.8m/s is the minimum required to ambulate independently and participate in community activities[12, 13]. Gait speeds of 0.4-0.8m/s have been associated with independently performing activities of daily living, but with limited community ambulation[13, 14]. Individuals with gait speeds <0.4m/s are likely to require assistance with performing some household activities and require full assistance (e.g., wheelchair use) for community activities.

For peer review only

Previous studies have reported estimates of clinically important differences in gait speed during the first year of recovery from a fracture and the characteristics of independent community ambulators[7, 15, 16]. Small, but meaningful improvements in gait speed, 0.10 to 0.17m/s, have been observed during recovery from hip fractures, and substantial improvements, 0.17 to 0.26m/s, have also been observed[16]. While proficient motor control and sound cognitive function have been associated with independent community ambulation, few studies have reported factors associated with changes in gait speed and discharge destination among older adults with orthopedic trauma related injuries[7, 15, 16].

This study had three objectives to be investigated in a sample of older adults admitted to a subacute hospital rehabilitation unit with orthopedic trauma related injuries. The first was to describe gait performance at admission and discharge from hospital rehabilitation. The second was to calculate the proportion of the sample who met a minimum gait speed threshold indicator for community ambulation at discharge and MCID (minimal clinically important difference) for change in gait speed during inpatient hospital rehabilitation. The third objective was to examine which patient and clinical factors were associated with a) change in gait speed, b) gait speed at discharge and c) discharge to a residential care facility (interim care, hostel or residential aged care facilities).

## Methods

### Design

A longitudinal cohort study with two assessment points was conducted.

### Participants and settings

Participants were patients, aged >=60 years, who presented to a tertiary hospital facility with fracture-related injuries that required an inpatient admission. At the participating hospital patients typically receive acute management of their fracture (e.g., reduction and stabilization) and acute inpatient care in a specialized orthopedics unit. Frail older adults who are not able to safely return home following their inpatient admission may receive further subacute inpatient rehabilitation within the hospital. This participating subacute hospital rehabilitation unit was a 76-bed facility providing comprehensive geriatric medicine and multidisciplinary therapy. This included weekday rehabilitative therapies (e.g., physical therapy), nurse supervised mobility (e.g., walking from the bedside to a ward-based dining area for meals for patients with appropriate mobility) and weekly case conferencing within the multidisciplinary team.

Patients were not eligible for inclusion if they were admitted for non-orthopedic traumatic injuries or for injuries that included serious neurological insults (e.g., spinal cord injury), or were transferred to another hospital without completing subacute inpatient hospital rehabilitation at the participating facility. Patients who died during their inpatient stay were also excluded.

**Procedures**

Patients admitted for subacute inpatient hospital rehabilitation at the participating hospital routinely complete assessments within 24 hours of their admission to the rehabilitation unit or prior to their discharge from the rehabilitation unit. However, on occasions of admission or discharge on weekend days, these assessments may be completed up to 72 hours of admission or prior to discharge. As part of these two assessments, gait performance was assessed by

For peer review only

their treating physiotherapists using the timed 10m walk test[17-19].

*Gait speed assessment*

The timed 10m walk test was used to assess patients' walking ability over 14m, with the middle 10m timed using a stopwatch. In the present study, treating physiotherapists followed standardized testing procedures[17-19], including instructions to walk at a comfortable and safe pace and permitting the use of a mobility aid (e.g., a walking frame). Patients unable to complete the test without physical assistance of another person were considered unable to complete the test.

*Clinical and patient characteristics*

Clinical and patient characteristics collected at admission included age, sex and type of injury(s). At admission and discharge from the subacute inpatient rehabilitation unit, the use of a mobility aid was recorded, and two functional measures were completed: the Functional Independence Measure (FIM motor and FIM cognitive) by an occupational therapist trained in the FIM assessment procedures, and the Modified Elderly Mobility Scale by a physiotherapist[19, 20]. At discharge, the length of stay in the hospital rehabilitation unit was recorded. Three rehabilitation outcomes were of interest for this study: change in gait speed (between the admission and discharge assessment), gait speed at discharge and discharge to a residential aged care facility.

**Statistical Analysis**

For the purpose of describing gait performance, participants were classified into one of four categories: a) unable to complete the timed 10m walk test at admission and discharge; b) unable to complete the timed 10m walk test at admission, but able at discharge c) able to

For peer review only

complete the test at admission and discharge, and d) able to complete the test at admission, but unable at discharge. Frequency histograms illustrate the distribution of discharge gait speed and change in gait speed after excluding patients unable to complete the assessments at admission and discharge. Frequencies (and proportion) of patients who met the previously reported minimum threshold gait speed of 0.8m/s[18], which is indicative of ability to successfully ambulate in the community, were calculated. Also calculated were the frequencies and proportions of patients who experienced a change in gait speed that exceeded a previously reported estimate of MCID of 0.10m/s [21].

Three generalized linear models were used to examine patient and clinical characteristics associated with a) change in gait speed, b) discharge gait speed and c) whether the patient was discharged to a residential aged care facility (yes or no). The Gaussian family and identity link option were used for both gait speed models, while the binomial family and logit link option were used for the model examining discharge to a residential aged care facility. Patient and clinical characteristics included as independent variables in all models were patient age (continuous, in years), sex, length of stay (continuous, in days), type of injury (nine categories), admission gait speed (continuous, in m/s), and use of a mobility aid at admission (no/yes). In the discharge destination model three additional variables were included: admission Modified Elderly Modified Scale (MEMS) score, admission FIM cognitive score and admission FIM motor score.

Fifty-nine patients (7.9%) had missing data on at least one variable, with missing data considered likely to be missing at random. Multiple imputations by chained equations were used to enable inclusion of these patients in the modeling[22]. Multicollinearity was assessed using variance inflation factors and tolerance statistics. All were <5, indicating


For peer review only - http://bmjopen.bmj.com/site/about/guidelines.xhtml

multicollinearity was not present. Robust standard errors (Huber-White Sandwich Variance Estimator approach) were used to account for potential heteroscedasticity[23, 24].

For the aforementioned primary generalized linear models that examined a) change in gait speed and b) discharge gait speed, participants unable to complete the time 10m walk test were assigned a gait-speed of 0m/s. Sensitivity analyses were also conducted for these two generalized linear models. These sensitivity analyses excluded patients assigned a gait speed of 0m/s for the primary analyses due to being unable to complete the timed 10m walk test at both admission and discharge assessments. This was conducted to examine whether the exclusion of this subset of patients influenced the findings of the aforementioned linear models. All analyses were conducted using Stata 13 (StataCorp LP, College Station, TX, USA) and alpha was set at a 0.05 significance level.

## RESULTS

A total of 774 patients were initially eligible for inclusion (figure 1). Of these, 28 were not included: 22 (2.8%) patients were transferred to another hospital unit prior to assessment and six (0.8%) died. The characteristics of the remaining 746 patients (96.4%) are presented in Table 1. A summary of gait speed at each assessment and improvement in gait speed has been provided in Table 2 and supplementary figure S1. In short, most (76.4%) patients who were able to complete the test at both assessments improved their gait speed by more than MCID in gait of 0.10m/s[21, 25], while just 1.3% decreased by a clinically meaningful margin.



For peer review only - http://bmjopen.bmj.com/site/about/guidelines.xhtml

**Table 1: Patient and clinical characteristics**

| Characteristics | | |
|---|---|---|
| Age (mean, SD) | 82 | 8 |
| Gender (n, %) | | |
| Male | 211 | 28.3 |
| Female | 535 | 71.7 |
| Type of injury (n, %) | | |
| Neck of femur | 403 | 54.0 |
| Pelvic | 86 | 11.5 |
| Upper limb | 63 | 8.4 |
| Spinal | 56 | 7.5 |
| Femoral | 41 | 5.5 |
| Multiple | 38 | 5.1 |
| Other axial | 31 | 4.2 |
| Lower limb | 28 | 3.8 |
| Mobility aid at admission assessment (n, %) | | |
| Walking frame (four wheeled walker or hopper) | 611 | 82 |
| Stick (single, two single, four-point) | 49 | 6.6 |
| Wheelchair | 39 | 5.2 |
| Nil | 37 | 4.9 |
| Others(crutches) | 10 | 1.3 |
| Discharge destination (n, %) | | |
| Home | 614 | 82.3 |
| Residential aged care facility (high level care facility) | 78 | 10.4 |
| Hostel (low level care facility) | 32 | 4.3 |
| Interim | 22 | 3.0 |
| Length of stay in days (median, IQR) | 35 | 23-56 |
| Mobility aid at discharge assessment (n,%) | | |
| Walking frame (four wheeled walker or hopper) | 448 | 60.2 |
| Stick (single, two single, four-point) | 207 | 27.7 |
| Nil | 62 | 8.3 |
| Wheelchair | 19 | 2.5 |
| Others (crutches) | 10 | 1.3 |

For peer review only

**Table 2: Mean gait speed at admission and discharge**

| Ability to complete timed 10m walk at each assessment | n | Mean gait speed at admission (SD) | Mean gait speed at discharge (SD) |
|---|---|---|---|
| Unable at admission & discharge | 61 | 0 (-) | 0 (-) |
| Unable at admission & able at discharge | 303 | 0 (-) | 0.53 (0.22) |
| Able at admission & unable at discharge | 1 | 0.18 (-) | 0 (-) |
| Able at admission & discharge | 381 | 0.37 (0.21) | 0.64 (0.25) |

*- indicates SD not applicable as there was no variation in gait speed for that category*

**Factors associated with gait speed at discharge from hospital rehabilitation**

Patients with pelvic fractures ($p<0.01$) and multiple fractures ($p<0.01$) had faster gait speed at discharge than patients with neck of femur fractures (Table 3). Compared with female patients, male patients were more likely to have faster gait speed at discharge ($p=0.05$). Also, patients who used a mobility aid at admission were more likely to have walked slower at discharge compared with those who did not use a mobility aid ($p=0.05$).

**Factors associated with change in gait speed during inpatient hospital rehabilitation**

Patients with pelvic fractures ($p<0.01$) and multiple fractures ($p<0.01$) demonstrated greater improvement in gait speed than patients with neck of femur fractures (Table 3). Male gender ($p=0.05$) was associated with greater improvement in gait speed, while use of a mobility aid at admission was associated with less improvement in gait speed ($p=0.05$).

For peer review only - http://bmjopen.bmj.com/site/about/guidelines.xhtml

**Table 3: Summary of coefficients from two generalized linear models examining the association of patient and clinical factors with a) change in gait speed b) gait speed at discharge**

| Model dependent variable | Independent variables | Coefficient × 10 | 95% Confidence Interval | | *p* value |
|---|---|---|---|---|---|
| Discharge gait speed | Age | -0.01 | -0.04 | 0.05[a] | 0.13 |
| | Male gender | 0.43 | -0.01[a] | 0.87 | 0.05 |
| | Length of stay | -0.04[a] | -0.01 | 0.03[a] | 0.26 |
| | Type of injury | | | | |
| *(Wald chi square(12)= 362.90, p < 0.001)* | Neck of femur | Referent | | | |
| | Spinal | 0.09 | -0.43 | 0.62 | 0.73 |
| | Pelvic | 0.76 | 0.14 | 1.37 | < 0.01 |
| | Upper limb | -0.57 | -1.18 | 0.02 | 0.06 |
| | Multiple | 1.13 | 0.25 | 2.01 | < 0.01 |
| | Femoral | -0.29 | -1.06 | 0.47 | 0.45 |
| | Lower limb | 0.68 | -0.31 | 1.69 | 0.17 |
| | Other axial | 0.44 | -0.46 | 1.35 | 0.33 |
| | Gait speed[a] | 5.77 | 5.03 | 6.50 | <0.001 |
| | Use of mobility aid[a] | -0.93 | -1.89 | 0.01 | 0.05 |
| Change in gait speed | Age | -0.01 | -0.04 | 0.05[a] | 0.13 |
| | Male gender | 0.43 | -0.01[a] | 0.87 | 0.05 |
| | Length of stay | -0.04[a] | -0.01 | 0.03 | 0.25 |
| *(Wald chi square(12)= 208.81, p < 0.001)* | Type of injury | | | | |
| | Neck of femur | Referent | | | |
| | Spinal | 0.09 | -0.43 | 0.62 | 0.73 |
| | Pelvic | 0.76 | 0.14 | 1.37 | < 0.01 |
| | Upper limb | -0.57 | -1.18 | 0.02 | 0.06 |
| | Multiple | 1.13 | 0.25 | 2.01 | < 0.01 |
| | Femoral | -0.29 | -1.06 | 0.47 | 0.45 |
| | Lower limb | 0.68 | -0.31 | 1.69 | 0.17 |
| | Other axial | 0.44 | -0.46 | 1.35 | 0.33 |
| | Gait speed[a] | -4.22 | -4.96 | -3.49 | <0.001 |
| | Use of mobility aid[a] | -0.93 | -1.89 | 0.01 | 0.05 |

*a= coefficients have been multiplied by 100*

**Factors associated with discharge to a residential aged care facility**

Older age (p<0.001) and prolonged length of stay (p<0.001) were associated with a lower likelihood of being discharged home (Table 4). Participants with upper limb fractures were less likely to be discharged home (p<0.01) than those with neck of femur fractures. A lower

For peer review only

FIM cognitive score was associated with a greater likelihood of being discharged to a residential aged care facility (p<0.001).

**Table 4: Summary of odds ratios from a generalized linear model examining the association of patient and clinical factors with discharge to a residential aged care facility**

| Model dependent variable | Independent variables | Odds Ratio | 95% Confidence Interval | | *p* value |
|---|---|---|---|---|---|
| Discharge to a residential aged care facility | Age | 1.06 | 1.03 | 1.10 | < 0.001 |
| | Male gender | 0.98 | 0.58 | 1.67 | 0.95 |
| | Length of stay | 1.01 | 1.01 | 1.02 | < 0.001 |
| | Type of injury | | | | |
| | Neck of femur | Referent | | | |
| *(Wald chi square(15)= 7.84, p < 0.001)* | Spinal | 1.28 | 0.49 | 3.34 | 0.60 |
| | Pelvic | 0.80 | 0.36 | 1.76 | 0.59 |
| | Upper limb | 2.81 | 1.32 | 5.97 | < 0.01 |
| | Multiple | 0.20 | 0.02 | 1.55 | 0.12 |
| | Femoral | 2.02 | 0.85 | 4.82 | 0.11 |
| | Lower limb | 0.83 | 0.22 | 3.10 | 0.79 |
| | Other axial | 0.25 | 0.03 | 2.12 | 0.20 |
| | MEMs score[a] | 0.97 | 0.89 | 1.05 | 0.46 |
| | FIM cognitive score[a] | 0.89 | 0.86 | 0.92 | < 0.001 |
| | FIM motor score[a] | 0.98 | 0.96 | 1.00 | 0.15 |
| | Gait speed[a] | 0.50 | 0.09 | 2.60 | 0.41 |
| | Use of mobility aid[a] | 0.85 | 0.31 | 2.31 | 0.75 |

a=Functional measures assessed at admission

**Sensitivity analyses**

The sensivity analysis findings were consistent with those of the primary analyses of factors associated with discharge gait speed and change in gait speed (supplementary Table S1). The coefficient point estimates were similar to those in the primary analyses, and disrepancies between the primary and sensitivity analysis confidence intervals were of a small magnitude.

For peer review only

## Discussion

This study is the first to examine gait speeds at discharge from subacute hospital rehabilitation in specifically in patients with orthopedic trauma related injuries as well as to examine associations between patient and clinical characteristics with gait outcomes and discharge to residential aged care. The gait outcomes ought to be interpreted in the context of the high level of frailty present in the patient sample. The findings could be interpreted as positive with the majority (91.7%) of patients able to walk at least a small distance without assistance of another person at discharge, a commendable achievement given approximately half of the participants were unable to walk without assistance of another person at admission to rehabilitation. Furthermore, a (relatively) low proportion (10.4%) of participants was discharged to a residential aged care facility for ongoing high level nursing care. However, this study also highlighted that only 18% of participants achieved the previously reported 0.8m/s threshold indicator for ability to ambulate in community environments[18].

Previous reports have proposed that gait speeds above 1.0m/s are desirable and those lower than 0.5m/s are associated with poor function and health[26-28]. The slow gait speed observed at completion of inpatient rehabilitation in the present study is not an indictment of the inpatient model of care at the participating facility. It was encouraging that over three quarters of participants could be considered to have improved their gait speed by a clinically meaningful margin during their stay. The mean gait speed at discharge was consistent with a systematic review of prior reports from similar subacute hospital settings that included non-orthopedic patients[29]. However, the findings highlight the importance of planning for an integrated transition from hospital to community-based services in the initial post-hospitalization period for older people recovering from orthopedic trauma. This may include engagement with community-based therapies for patients who have potential to extend gains


For peer review only - http://bmjopen.bmj.com/site/about/guidelines.xhtml

attained during inpatient rehabilitation, as well as supportive care interventions that seek to maximize patients' quality of life and mitigate risk of adverse events. Prior research has highlighted the potential effectiveness (and cost-effectiveness) of community-based interventions for frail older adults recovering from injury and illness[30, 31].

Prior studies in the field have typically focused on patients recovering from hip fractures and have often emphasized risk of undesirable outcomes following hip fractures[32-34]. The inclusion of patients recovering from fractures affecting a range of body regions in the present study permitted novel contributions, particularly in the analytical models that examined factors associated with outcomes at discharge from inpatient rehabilitation. For example, patients recovering from upper limb injuries were more likely to be discharged to residential aged care facilities than patients recovering from hip fractures. At first, this finding may seem paradoxical given the extent of prior reporting of challenges and functional deficits faced by people recovering from hip fractures [35, 36], but it may be explained by the likely high level of frailty in patients requiring inpatient hospital rehabilitation following an upper-limb fracture. It is likely that most patients who present to hospital with an upper-limb fracture will be discharged soon after their fracture has been stabilized, and relatively few will require subacute inpatient rehabilitation. Of all older adults who sustain an upper limb injury, it is likely to be the most frail, complex or socially vulnerable who will require subacute inpatient hospital rehabilitation[37]. In comparison, a high proportion of patients who sustain a hip fracture may require subacute hospital rehabilitation regardless of their social circumstances or premorbid functioning to continue rehabilitative therapies in an effort to attain independence with mobility tasks prior to discharge from hospital.

For peer review only

Understanding factors associated with patients experiencing the greatest (or least) improvement in gait speed between admission and discharge offers insights useful for clinical teams, health service policy related to inpatient rehabilitation and future research in the field. In the present study the greatest improvements in gait speed were observed among patients with pelvic fractures or with multiple traumatic injuries, which was an encouraging finding as patients from these two diagnostic groups have received limited attention in prior research[29, 38]. It was also encouraging that patients with a slow baseline gait speed were among those to demonstrate the greatest improvement in gait speed during their inpatient rehabilitation, perhaps indicative of the fulfilment of (at least theoretical) potential for improvement that accompanies starting from a lower base than others in the cohort. However, not requiring the use of a mobility aid at admission was associated with greater improvement in gait speed. It is plausible that patients not requiring a mobility aid at admission to the rehabilitation unit had lower levels of pre-morbid physical frailty and were more readily able to return to higher gait speeds by discharge than peers in their cohort.

**Strengths and limitations**

A strength of this study was the inclusion of a range of orthopedic injury types present among older adults who were receiving hospital rehabilitation. Second, the inclusion of consecutive admissions meeting the inclusion criteria, with a relatively low rate of missing assessments reduced the risk of sampling bias. One limitation of this approach was that clinical variables were limited to those recorded for all participants during their routine clinical assessment. This meant that height was not available as a potential covariate for adjustment in the generalized linear models. Another important limitation of the study was that it was conducted at a single geographical location in an industrialized nation in a subacute hospital rehabilitation setting. It is possible that older adults recovering from orthopedic injuries in

For peer review only

dissimilar societies or healthcare systems may not have had comparable gait performances as patients in the present sample. The study design was appropriate for addressing the intended aims, but it did not allow any conclusions to be drawn regarding the effectiveness (or otherwise) of the model of care received by patients in improving gait speed in comparison to other models of care. This topic was considered beyond the scope of the present study, but future investigation of the optimal type and intensity of rehabilitation interventions for improving gait among older adults recovering orthopedic injuries remains a priority for research.

## Conclusion

Most patients were able to complete the 10m walk test without assistance of another person at discharge, but few were likely to be able to ambulate in community environments. The greatest improvements in gait speed were observed among patients with pelvic fractures and those with multiple traumatic injuries. Also noteworthy was that patients receiving hospital-based rehabilitation following injuries affecting upper limbs were more likely to be discharged to residential aged care facilities than patients recovering from hip fractures. Overall, the findings highlight the importance of planning for an integrated transition from hospital to community-based services in the initial post-hospital rehabilitation period. This may include engagement with community-based rehabilitative therapies or supportive care interventions to improve quality of life and mitigate risk of further adverse health events.

For peer review only

## Acknowledgments

*Funding Source*: The study was supported in part by the Health Practitioner Research Support Scheme (Queensland Department of Health). SMM is supported by a National Health and Medical Research Council (of Australia) fellowship

*Author Contributions:* SSK, PV, SMM: obtaining funding and acquisition of data. SAM, SMM, KCH: study design, analysis and interpretation of data. SAM, SMM: drafting of the manuscript. All authors: interpretation of findings, revision of manuscript, approval of final version

.

*No competing interests*: Authors have read and understood BMJ policy on declaration of interests and declare that we have no competing interests.

*Study funding:* The Department of Health Queensland financially supported the study but had no role in the research design, reporting or publishing decision

*Data sharing:* No additional data are available, though details on statistical analysis are available from the corresponding author on request

For peer review only



For peer review only - http://bmjopen.bmj.com/site/about/guidelines.xhtml

## References

[1] Wiktorowicz M, Goeree R, Papaioannou A, Adachi JD, Papadimitropoulos E. Economic implications of hip fracture: health service use, institutional care and cost in Canada. *Osteoporos Int*. 2001;12:271-278.

[2] Kawryshanker S, Raymond W, Ingram K, Inderjeeth CA. Effect of frailty on functional gain, resource utilisation, and discharge destination: an observational prospective study in a GEM Ward. *Curr Gerontol Geriatr Res*. 2014;2014.

[3] Pretto M, Spirig R, Kaelin R, Muri-John V, Kressig R, Suhm N. Outcomes of elderly hip fracture patients in the Swiss healthcare system. *Swiss Med Wkly*. 2010;140:w13086.

[4] Levers MJ, Estabrooks CA, Ross Kerr JC. Factors contributing to frailty: literature review. *J Adv Nurs.* 2006;56:282-291.

[5] Röder F, Schwab M, Aleker T, Mörike K, Thon KP, Klotz U. Proximal femur fracture in older patients–rehabilitation and clinical outcome. *Age Ageing*. 2003;32:74-80.

[6] Ostir GV, Berges I, Kuo Y-F, Goodwin JS, Ottenbacher KJ, Guralnik JM. Assessing gait speed in acutely ill older patients admitted to an acute care for elders hospital unit. *Arch Intern Med*. 2012;172:353-358.

[7] Hardy SE, Perera S, Roumani YF, Chandler JM, Studenski SA. Improvement in usual gait speed predicts better survival in older adults. *J Am Geriatr Soc*. 2007;55:1727-1734.

[8] Barthuly AM, Bohannon RW, Gorack W. Gait speed is a responsive measure of physical performance for patients undergoing short-term rehabilitation. *Gait Posture.* 2012;36:61-64.

[9] Barthuly AM, Bohannon RW, Gorack W. Limitations in gait speed persist at discharge from subacute rehabilitation. *J Phys Ther Sci.* 2013;25:891-893.

[10] Potter JM, Evans AL, Duncan G. Gait speed and activities of daily living function in geriatric patients. *Arch Phys Med Rehabil.* 1995;76:997-999.

For peer review only - http://bmjopen.bmj.com/site/about/guidelines.xhtml

[11] Purser JL, Weinberger M, Cohen HJ, Pieper CF. Walking speed predicts health status and hospital costs for frail elderly male veterans. *J Rehabil Res Dev.* 2005;42:535.

[12] Andrews AW, Chinworth SA, Bourassa M, Garvin M, Benton D, Tanner S. Update on distance and velocity requirements for community ambulation. *J Geriatr Phys Ther.* 2010;33:128-134.

[13] Fritz S, Lusardi M. White paper:"walking speed: the sixth vital sign". *J Geriatr Phys Ther.* 2009;32:2-5.

[14] Lord SE, McPherson K, McNaughton HK, Rochester L, Weatherall M. Community ambulation after stroke: how important and obtainable is it and what measures appear predictive? *Arch Phys Med Rehabil.* 2004;85:234-239.

[15] Lord SE, Weatherall M, Rochester L. Community ambulation in older adults: which internal characteristics are important? *Arch Phys Med Rehabil.* 2010;91:378-383.

[16] Alley DE, Hicks GE, Shardell M, Hawkes W, Miller R, Craik RL, et al. Meaningful improvement in gait speed in hip fracture recovery. *J Am Geriatr Soc.* 2011;59:1650-1657.

[17] Stineman MG, Shea JA, Jette A, Tassoni CJ, Ottenbacher KJ, Fiedler R, et al. The Functional Independence Measure: tests of scaling assumptions, structure, and reliability across 20 diverse impairment categories. *Arch Phys Med Rehabil.* 1996;77:1101-8.

[18] Van Kan GA, Rolland Y, Andrieu S, Bauer J, Beauchet O, Bonnefoy M, et al. Gait speed at usual pace as a predictor of adverse outcomes in community-dwelling older people an International Academy on Nutrition and Aging (IANA) Task Force. J *Nutr Health Aging*. 2009;13:881-9.

[19] Kuys SS, Brauer SG. Validation and reliability of the modified elderly mobility scale. *Australas J Ageing*. 2006;25:140-4.

For peer review only



For peer review only - http://bmjopen.bmj.com/site/about/guidelines.xhtml

[20] Kidd D, Stewart G, Baldry J, Johnson J, Rossiter D, Petruckevitch A, et al. The Functional Independence Measure: a comparative validity and reliability study. *Disabil Rehabil*. 2009.

[21] Bohannon RW, Glenney SS. Minimal clinically important difference for change in comfortable gait speed of adults with pathology: a systematic review. *J Eval Clin Pract*. 2014;20:295-300.

[22] Royston P, White IR. Multiple imputation by chained equations (MICE): implementation in Stata. *J Stat Softw*. 2011;45:1-20.

[23] Huber PJ. The behavior of maximum likelihood estimates under nonstandard conditions. *Proceedings of the fifth Berkeley symposium on mathematical statistics and probability* 1967; 221-33.

[24] White H. A heteroskedasticity-consistent covariance matrix estimator and a direct test for heteroskedasticity. *Econometrica*. 1980:817-838.

[25] Palombaro KM, Craik RL, Mangione KK, Tomlinson JD. Determining meaningful changes in gait speed after hip fracture. *Phys Ther*. 2006;86:809-816.

[26] Gard SA. Use of quantitative gait analysis for the evaluation of prosthetic walking performance. JPO: *J Prosthet Orthot*. 2006;18:P93-P104.

[27] Mendelsohn ME, Leidl DS, Overend TJ, Petrella RJ. Specificity of functional mobility measures in older adults after hip fracture: a pilot study. *Am J Phys Med Rehabil*. 2003;82:766-774.

[28] Robinett CS, Vondran MA. Functional Ambulation Velocity and Distance Requirements in Rural and Urban Communities A Clinical Report. *Phys Ther*. 1988;68:1371-1373.

[29] Peel NM, Kuys SS, Klein K. Gait speed as a measure in geriatric assessment in clinical settings: a systematic review. *J Gerontol Series A Biol Sci Med Sci*. 2013;68:39-46.

For peer review only

[30] Leveille SG, Wagner EH, Davis C, Grothaus L, Wallace J, LoGerfo M, et al. Preventing disability and managing chronic illness in frail older adults: A randomized trial of a community-based partnership with primary care. *J Am Geriatr Soc.* 1998;46:1191-1198.

[31] Hausdorff JM, Rios DA, Edelberg HK. Gait variability and fall risk in community-living older adults: a 1-year prospective study. *Arch Phys Med Rehabil.* 2001;82:1050-1056.

[32] Cornwall R, Gilbert MS, Koval KJ, Strauss E, Siu AL. Functional outcomes and mortality vary among different types of hip fractures: a function of patient characteristics. *Clin Orthop Relat Res.* 2004:64.

[33] Afilalo J, Eisenberg MJ, Morin J-F, Bergman H, Monette J, Noiseux N, et al. Gait speed as an incremental predictor of mortality and major morbidity in elderly patients undergoing cardiac surgery. *J Am Coll Cardiol.* 2010;56:1668-1676.

[34] Atkinson HH, Rosano C, Simonsick EM, Williamson JD, Davis C, Ambrosius WT, et al. Cognitive function, gait speed decline, and comorbidities: the health, aging and body composition study. *J Gerontol Series A Biol Sci Med Sci.* 2007;62:844-850.

[35] Koval KJ, Skovron ML, Aharonoff GB, Meadows SE, Zuckerman JD. Ambulatory ability after hip fracture: a prospective study in geriatric patients. *Clin Orthop Relat Res.* 1995:150-159.

[36] Dennett AM, Taylor NF, Mulrain K. Community ambulation after hip fracture: completing tasks to enable access to common community venues. *Disabil Rehabil.* 2012;34:707-714.

[37] Lübbeke A, Stern R, Grab B, Herrmann F, Michel J-P, Hoffmeyer P. Upper extremity fractures in the elderly: consequences on utilization of rehabilitation care. *Aging Clin Exp Res.* 2005;17:276-280.

For peer review only

[38] Kwakkel G, Wagenaar RC. Effect of duration of upper-and lower-extremity rehabilitation sessions and walking speed on recovery of interlimb coordination in hemiplegic gait. *Phys Ther*. 2002;82:432-448.

For peer review only

## Legends

**Figure 1** Participant flow diagram

**Supplementary figure S1** Frequency of discharge gait speed and change of gait speed

**Supplementary material S1-** Sensitivity analyses coefficients from two generalized linear models examining the association of patient and clinical factors with a) change in gait speed and b) gait speed at discharge

For peer review only

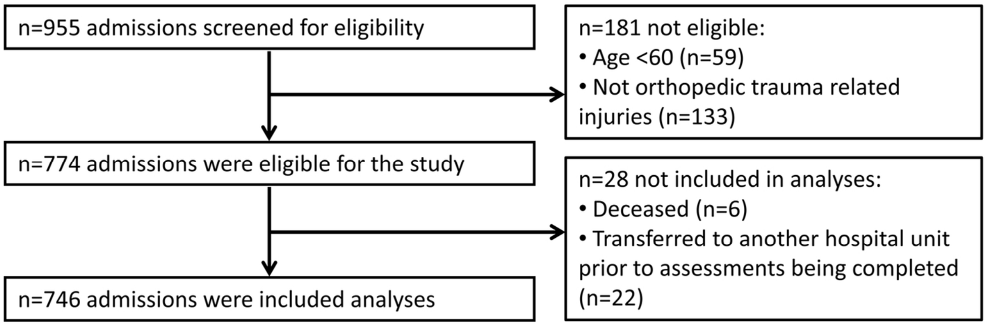


Figure 1. Participant flow diagram

64x21mm (600 x 600 DPI)

peer review only

Supplementary Table S1

**Sensitivity analyses coefficients from two generalized linear models examining the association of patient and clinical factors with a) change in gait speed and b) gait speed at discharge**

| **Model dependent variable** | **Independent variables** | **Coefficient × 10** | **95% Confidence Interval** | | ***p* value** |
|---|---|---|---|---|---|
| Discharge gait speed | Age | -0.01 | -0.03 | 0.00 | 0.24 |
| | Male gender | 0.54 | 0.14 | 0.95 | < 0.01 |
| | Length of stay | -0.01 | -0.06 | 0.08[a] | 0.76 |
| | Type of injury | | | | |
| *(Wald chi square(12)= 241.58, p < 0.001)* | Neck of femur | Referent | | | |
| | Spinal | 0.13 | -0.34 | 0.61 | 0.58 |
| | Pelvic | 0.69 | 0.12 | 1.25 | < 0.01 |
| | Upper limb | -0.32 | -0.91 | 0.26 | 0.27 |
| | Multiple | 1.08 | 0.28 | 1.89 | < 0.01 |
| | Femoral | -0.16 | -0.83 | 0.50 | 0.62 |
| | Lower Limb | 0.18 | -0.79 | 1.17 | 0.71 |
| | Other axial | 0.15 | -0.73 | 1.10 | 0.73 |
| | Gait speed | 4.54 | 3.81 | 5.27 | <0.001 |
| | Use of mobility aid | -0.95 | -1.86 | -0.04 | 0.03 |
| Change in gait speed | Age | -0.01 | -0.03 | 0.00 | 0.14 |
| | Male gender | 0.51 | -0.00 | 0.87 | < 0.01 |
| | Length of stay | 0.04 | -0.06 | 0.08[a] | 0.91 |
| *(Wald chi square(12)= 344.77, p < 0.001)* | Type of injury | | | | |
| | Neck of femur | Referent | | | |
| | Spinal | -0.13 | -0.34 | 0.61 | 0.58 |
| | Pelvic | 0.69 | 0.12 | 1.25 | < 0.01 |
| | Upper limb | -0.32 | -0.91 | 0.26 | 0.27 |
| | Multiple | 1.08 | 0.28 | 1.89 | < 0.01 |
| | Femoral | -0.16 | -0.83 | 0.50 | 0.62 |
| | Lower Limb | 0.18 | -0.79 | 1.17 | 0.71 |
| | Other axial | 0.15 | -0.73 | 1.04 | 0.73 |
| | Gait speed | -5.45 | -6.18 | -4.72 | <0.001 |
| | Use of mobility aid | -0.95 | -1.86 | 0.04 | 0.03 |

a= coefficients have been multiplied x $10^2$

For peer review only

For peer review only - http://bmjopen.bmj.com/site/about/guidelines.xhtml

For peer review only

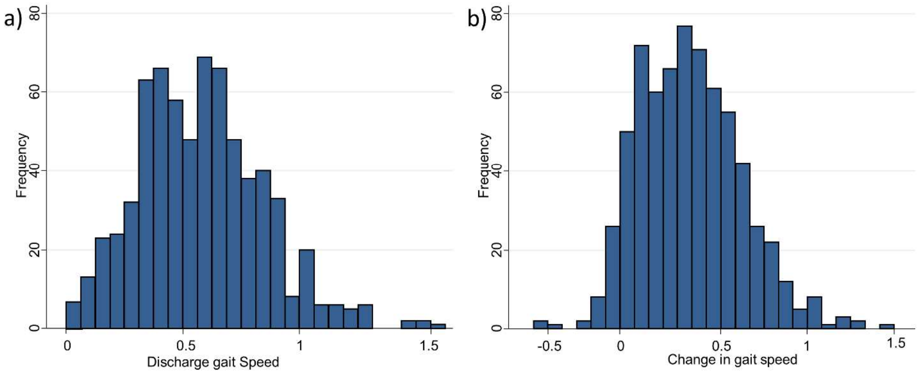


254x190mm (300 x 300 DPI)

ew only

**STROBE 2007 (v4) Statement—Checklist of items that should be included in reports of *cohort studies***

| Section/Topic | Item # | Recommendation | Reported on page # |
|---|---|---|---|
| **Title and abstract** | 1 | (*a*) Indicate the study's design with a commonly used term in the title or the abstract | 1-2 |
| | | (*b*) Provide in the abstract an informative and balanced summary of what was done and what was found | 2-3 |
| **Introduction** | | | |
| Background/rationale | 2 | Explain the scientific background and rationale for the investigation being reported | 4-5 |
| Objectives | 3 | State specific objectives, including any prespecified hypotheses | 5 |
| **Methods** | | | |
| Study design | 4 | Present key elements of study design early in the paper | 5 |
| Setting | 5 | Describe the setting, locations, and relevant dates, including periods of recruitment, exposure, follow-up, and data collection | 6 |
| Participants | 6 | (*a*) Give the eligibility criteria, and the sources and methods of selection of participants. Describe methods of follow-up | 6-7 |
| | | (*b*) For matched studies, give matching criteria and number of exposed and unexposed | |
| Variables | 7 | Clearly define all outcomes, exposures, predictors, potential confounders, and effect modifiers. Give diagnostic criteria, if applicable | 7 |
| Data sources/ measurement | 8* | For each variable of interest, give sources of data and details of methods of assessment (measurement). Describe comparability of assessment methods if there is more than one group | 7 |
| Bias | 9 | Describe any efforts to address potential sources of bias | 6 |
| Study size | 10 | Explain how the study size was arrived at | 6 |
| Quantitative variables | 11 | Explain how quantitative variables were handled in the analyses. If applicable, describe which groupings were chosen and why | 7 |
| Statistical methods | 12 | (*a*) Describe all statistical methods, including those used to control for confounding | 7-8 |
| | | (*b*) Describe any methods used to examine subgroups and interactions | 7 |
| | | (*c*) Explain how missing data were addressed | 8 |
| | | (*d*) If applicable, explain how loss to follow-up was addressed | 8 |
| | | (*e*) Describe any sensitivity analyses | 9 |
| **Results** | | | |

For peer review only

| | | | |
|---|---|---|---|
| Participants | 13* | (a) Report numbers of individuals at each stage of study—eg numbers potentially eligible, examined for eligibility, confirmed eligible, included in the study, completing follow-up, and analysed | Figure 1<br>Page 9 |
| | | (b) Give reasons for non-participation at each stage | Figure1 |
| | | (c) Consider use of a flow diagram | Figure 1 |
| Descriptive data | 14* | (a) Give characteristics of study participants (eg demographic, clinical, social) and information on exposures and potential confounders | Table 1<br>Page 9 |
| | | (b) Indicate number of participants with missing data for each variable of interest | Table 1 |
| | | (c) Summarise follow-up time (eg, average and total amount) | Table 1, Page 9 |
| Outcome data | 15* | Report numbers of outcome events or summary measures over time | Table 2. Supplementary figure S1, Page9 |
| Main results | 16 | (*a*) Give unadjusted estimates and, if applicable, confounder-adjusted estimates and their precision (eg, 95% confidence interval). Make clear which confounders were adjusted for and why they were included | Table 3 & Table 4<br>Page 11-13 |
| | | (*b*) Report category boundaries when continuous variables were categorized | |
| | | (*c*) If relevant, consider translating estimates of relative risk into absolute risk for a meaningful time period | |
| Other analyses | 17 | Report other analyses done—eg analyses of subgroups and interactions, and sensitivity analyses | Supplementary table S1, Page 13 |
| **Discussion** | | | |
| Key results | 18 | Summarise key results with reference to study objectives | 14-17 |
| **Limitations** | | | |
| Interpretation | 20 | Give a cautious overall interpretation of results considering objectives, limitations, multiplicity of analyses, results from similar studies, and other relevant evidence | 16 |
| Generalisability | 21 | Discuss the generalisability (external validity) of the study results | 16 |
| **Other information** | | | |
| Funding | 22 | Give the source of funding and the role of the funders for the present study and, if applicable, for the original study on which the present article is based | 18 |

*Give information separately for cases and controls in case-control studies and, if applicable, for exposed and unexposed groups in cohort and cross-sectional studies.

For peer review only

**Note:** An Explanation and Elaboration article discusses each checklist item and gives methodological background and published examples of transparent reporting. The STROBE checklist is best used in conjunction with this article (freely available on the Web sites of PLoS Medicine at http://www.plosmedicine.org/, Annals of Internal Medicine at http://www.annals.org/, and Epidemiology at http://www.epidem.com/). Information on the STROBE Initiative is available at www.strobe-statement.org.

For peer review only
